# SNP Array for Small-Shrimp (Genus *Acetes*) Origin Determination Using Machine Learning

**DOI:** 10.3390/foods13132087

**Published:** 2024-07-01

**Authors:** Eun Soo Noh, Mi Nan Lee, Chun-Mae Dong, Jungwook Park, Hyo Sun Jung, Woo-Jin Kim, Young-Ok Kim

**Affiliations:** Biotechnology Research Division, National Institute of Fisheries Science, 216, Gijanghaean-ro, Gijang-eup, Gijang-gun, Busan 46083, Republic of Korea; minan012@naver.com (M.N.L.); ehdcnsao@aver.com (C.-M.D.); jjuwoogi@korea.kr (J.P.); jhs3010@korea.kr (H.S.J.); wj2464@korea.kr (W.-J.K.); yobest12@korea.kr (Y.-O.K.)

**Keywords:** small shrimp, machine learning, origin, single-nucleotide polymorphism

## Abstract

Accurate origin determination of seafood is crucial for consumer trust and safety. This study was performed to develop a machine learning-based single-nucleotide polymorphism (SNP) analysis technique to determine the origin of *Acetes* species in salted small-shrimp products. Mitochondrial DNA (COI and 16S rRNA) analysis revealed genetic variations among species and origins. Eight candidate SNPs were identified, six of which were developed into markers for genotyping analysis. Using the developed markers, an SNP array was created and SNP data from salted small-shrimp samples were obtained. Machine learning analysis using a supervised learning algorithm achieved 100% accuracy in classifying the origin of *Acetes* based on SNP data. This method offers a reliable method for regulatory bodies to combat food fraud and ensure product integrity. The approach can be further improved by expanding the data set to encompass a wider range of species and origins. This study highlights the potential of SNP analysis and machine learning for ensuring seafood authenticity and promoting sustainable practices.

## 1. Introduction

Issues related to the safety and quality of seafood have emerged, with consumers increasingly paying more attention to the origin of products due to concerns about health and food safety [1]. Knowing the origin of seafood allows consumers to make informed choices and avoid potential health hazards related to the use of antibiotics in aquaculture, pollution in waterways, and mislabeling [2]. Clearly indicating the origin of food ensures trust in the product, reflecting the natural environment, production technologies, and conditions involved in food production [3]. For example, Norwegian salmon may be perceived as superior due to specific production methods, environmental factors, or regulations [4]. Knowing the origin of foods allows producers to charge a premium for these desired qualities. Unfortunately, the mislabeling of seafood is an ongoing problem. Unscrupulous sellers may pass off lower quality or less desirable species as more valuable alternatives, which not only deceives consumers but also undermines trust in the seafood industry. Accurate origin determination helps to combat food fraud and ensure consumers receive the products they have paid for. Furthermore, producers in each country or region strive to enhance the competitiveness of their seafood and food products by emphasizing their origin [5].

Consumers often place high trust in foods from certain countries, including domestic production [6]. The mislabeling of seafood of lower quality as more desirable species poses risks to consumer health due to unreliable production environments or processes [7]. To prevent such issues, ensure consumer safety, and establish order in distribution, research on origin determination of various seafood products is required.

In the context of Korean cuisine, where small shrimp (*Acetes* species) are consumed in large quantities and play a significant role in traditional dishes like kimchi, ensuring their authenticity and origin is particularly crucial [8]. In particular, the rising demand for these shrimp has led to a decline in domestic catches and a subsequent price increase, creating an opportunity for fraudulent mislabeling of imported shrimp as domestic [9]. Accurate species and origin determination is essential to maintain consumer confidence and uphold the quality of domestic products.

There are two main approaches to determining the origin of seafood. The first approach leverages genetic differences within the same species resulting from geographical isolation among countries. Population genetic analysis plays a crucial role in such studies. By studying these genetic variations, populations from different regions can be distinguished; for example, studies can use microsatellite markers and genetic variations to determine the origin of small shrimp (*Acetes chinensis*) and Manila clams (*Ruditapes philippinarum*) [9,10]. The second approach focuses on identifying the species of seafood, which becomes particularly relevant when dealing with species that are not native to a particular country or that closely resemble others. Here, techniques such as multiplex PCR analysis are used to differentiate between domestic and imported seafood or to identify non-native species that may be present. For instance, studies have used multiplex PCR to analyze sole (*Cynoglossus*), croakers (*Larimichthys*, *Atrobucca*, and *Pseudotolithus*), and loaches (*Misgurnus* and *Paramisgurnus*) [11,12,13].

Species identification is fundamental to taxonomic research and is a crucial element in studies of ecology and evolution [14]. Accurate species identification underpins research on biodiversity, the monitoring of endangered populations, and aquaculture and fisheries research [15,16]. Morphological and molecular biological methods are generally used for identification, with molecular methods complementing the limitations of morphological classification and enhancing accuracy [17]. One commonly used molecular method involves the analysis of single-nucleotide polymorphisms (SNPs), genetic variations where a single nucleotide differs between members of the same or different species. These variations in genes serve as effective genetic markers in both animals and plants, providing insight into population compositions [18]. SNP analysis technology has applications in a number of fields, including forensic research and studies of biological evolution [18,19].

Research on SNPs can be significantly enhanced when combined with machine learning (ML), a rapidly growing field of computer science that can uncover complex correlations hidden in large data sets obtained by next-generation sequencing [20]. ML uses a range of algorithms to generate models from existing data, facilitating pattern recognition and classification, thus yielding predictions. These algorithms can be broadly divided into supervised learning, which involves training with well-classified objects to predict the classification of new objects, and unsupervised learning, which involves classifying provided objects without a training process [21]. Such ML analyses have applications in various biological fields, such as pattern recognition in coding sequences, the identification of biomarkers, and the exploration of disease genes [22,23].

In this study, an ML-based SNP analysis technique was developed and evaluated to accurately discriminate the origin of small shrimp. By leveraging the power of ML algorithms, complex correlations within mitochondrial DNA data sets were uncovered to facilitate efficient pattern recognition and classification, ultimately leading to robust predictive models for determining the geographical origin of small-shrimp samples. This integrated approach combines the high accuracy of SNP analysis with the computational efficiency of ML, offering a promising solution for addressing challenges related to seafood mislabeling and ensuring the authenticity of small-shrimp products.

## 2. Materials and Methods

### 2.1. Sample Preparation

Salted shrimp samples were purchased from a local market for genetic analysis. The origin of each sample was labeled on the product packaging (Table 1). Genomic DNA was extracted using a DNeasy Blood & Tissue Kit (Qiagen Inc., Toronto, ON, Canada) in accordance with the manufacturer’s protocol. Briefly, 20 mg of muscle tissue was lysed with 180 μL of ATL buffer and 20 μL of proteinase K at 56 °C for 1 h. Then, 180 μL of AL buffer and 180 μL of ethanol (99%) were added, the sample was mixed, and the supernatant was transferred to a column and centrifuged at 8000 rpm. The DNA was washed twice with 300 μL of AW1 buffer and an equal volume of AW2 buffer, and then eluted with TE buffer. The extracted genomic DNA was visualized on a 1% (*w*/*v*) agarose gel and quantified by spectrophotometry (NanoVue; GE Healthcare, Fairfield, NJ, USA).

### 2.2. Sequence Analyzing

Mitochondrial DNA (mtDNA) barcode regions—the cytochrome oxidase subunit I (COI) gene and 16S ribosomal RNA (16S rRNA) gene—were amplified and sequenced to identify the species and compare interspecific genetic variation. The following universal primers were used: LCO1490 (5′-GGT CAA CAA ATC ATA AAG ATA TTG G-3′) and HCO2198 (5′-TAA ACT TCA GGG TGA CCA AAA AAT CA-3′) for COI; and 16Sar (5′-CGC CTG TTT ATC AAA AAC AT-3′) and 16Sbr (5′-CCG GTC TGA ACT CAG ATC ATG T-3′) for 16S rRNA [24,25]. PCR was performed in a mixture consisting of 1 μL of genomic DNA, 2 μL of 10× Ex Taq buffer, 1.6 μL of dNTP mixture (2.5 mM), 1 μL of forward primer (10 pmol), 1 μL of reverse primer (10 pmol), and 5 units of Ex Taq polymerase (Takara Bio, Otsu, Japan) in a total volume of 20 μL. PCR amplification was performed using an ABI Veriti Fast Thermal Cycler (Applied Biosystems, Foster City, CA, USA) with an initial denaturation step at 94 °C for 10 min followed by 32 cycles of denaturation at 94 °C for 30 s, annealing at 58 °C for 30 s, and extension at 72 °C for 1 min, with a final extension at 72 °C for 7 min. The PCR products were visualized on a 1% (*w*/*v*) agarose gel to confirm amplification, and then sequenced using a BigDye Terminator v3.1 Cycle Sequencing Kit (Applied Biosystems) and an ABI3730XL sequencer.

### 2.3. SNP Discovery

Sequence data produced from the samples were assembled and aligned using SeqMan Pro v17.3.0 (DNA Star, Madison, WI, USA). Intraspecific genetic variation and haplotypes by group were analyzed using DnaSP v5.10.01 [26]. Single-nucleotide variants that showed differences between each group were then identified. Intraspecific genetic distances were analyzed using the Kimura-2-parameter model in MEGA 10 and summarized in a phylogenetic tree using the neighbor-joining (NJ) method [27].

### 2.4. Designing SNP Genotyping Array

Primers for the eight selected SNPs were designed using the Fluidigm D3 Assay Design Tool (http://d3.standardbio.com/; accessed on 11 October 2023) along with their flanking sequences. The primers and Integrated Fluidic Circuit (96.96 IFC) array were ordered from Fluidigm (Fluidigm Corp., San Francisco, CA, USA). To test the performance of the manufactured SNP panel, 96 randomly selected small-shrimp samples that had been used for sequence analysis were analyzed.

### 2.5. Machine Learning Analysis

SNP information obtained from each salted shrimp product was used as training data for the ML analysis to enable rapid result interpretation and improve accuracy. The ML analysis was performed using WEKA 3.8.5 with the Random Forest algorithm, a supervised learning method chosen for its ability to handle high-dimensional data, such as SNP information, and its robustness against overfitting [28]. Where a well-trained model was used to analyze the provided data, the accuracy of the developed ML workflows was evaluated using SNP information from random samples obtained using the Fluidigm array as test data.

## 3. Results

### 3.1. Sequencing Analysis

**Figure 1:**
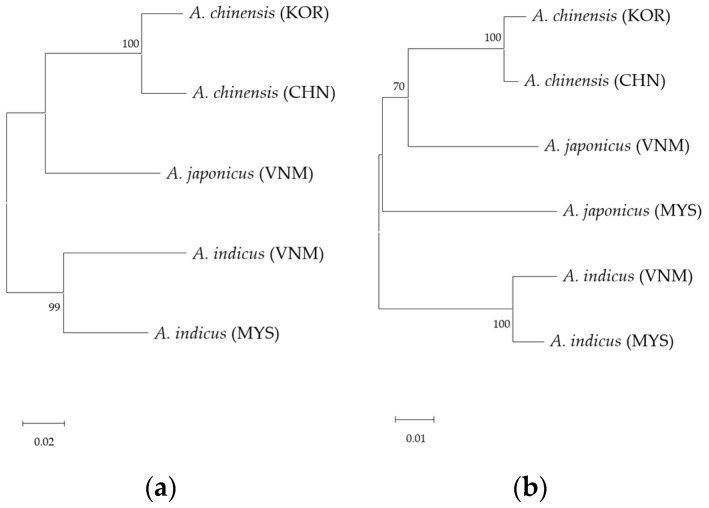
Phylogenetic analysis of small shrimp from Korea (KOR), China (CHN), Vietnam (VNM), and Malaysia (MYS) using neighbor-joining method. (**a**) Mitochondrial COI region; (**b**) mitochondrial 16S rRNA region.

Genetic information was obtained from up to 48 samples selected from 14 salted small-shrimp products labeled as originating from Korea, China, Vietnam, and Malaysia. The COI and 16S rRNA gene regions were obtained from the products labeled as coming from Korea. NCBI BLAST analysis revealed that both gene regions showed 100% identity to *A. chinensis* MK941905.1 in the COI region and 99.8% identity to MK928508.1 in the 16S rRNA region. For shrimp paste labeled as coming from China, the COI and 16S rRNA regions were also obtained, and both regions showed 100% identity to *A. chinensis* MK941908.1 and MK928507.1. Although both origins were identified as the same species, *A. chinensis* from Korea and China exhibited genetic differences of 3.95% in the COI region and 0.96% in the 16S rRNA region (Figure 1).

One of the salted small-shrimp products labeled as coming from Vietnam was identified as *A. japonicus*, showing 100% identity to MZ412582.1 in the COI region and 99.42% identity to MZ412582.1 in the 16S rRNA region. Another product from the same origin was identified as *Acetes indicus*, exhibiting 100% identity to OP420230.1 in the COI region and 99.42% identity to MK245778.1 in the 16S rRNA region. The salted small-shrimp product labeled as coming from Malaysia contained a mixture of *A. japonicus* and *A. indicus*. *A. japonicus* showed 99.36% identity to MK412582.1 in the 16S rRNA region, but the COI gene could not be obtained. In addition, *A. indicus* exhibited 100% identity to HQ630436.1 in the COI region and 97.31% identity to MK245778.1 in the 16S rRNA region. However, *A. japonicus* from Vietnam and Malaysia samples showed a genetic difference of 8.43% in the 16S rRNA gene region. Furthermore, *A. indicus* from Vietnam and Malaysia samples exhibited genetic differences of 9.81% in the COI region and 2.12% in the 16S rRNA region.

### 3.2. SNP Discovery

**Figure 2:**
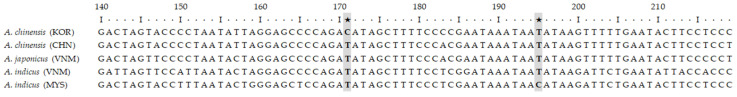
Candidate SNP marker sites selected from mitochondrial COI region. The asterisks in the figure indicate the polymorphic sites for origin determination.

**Figure 3:**
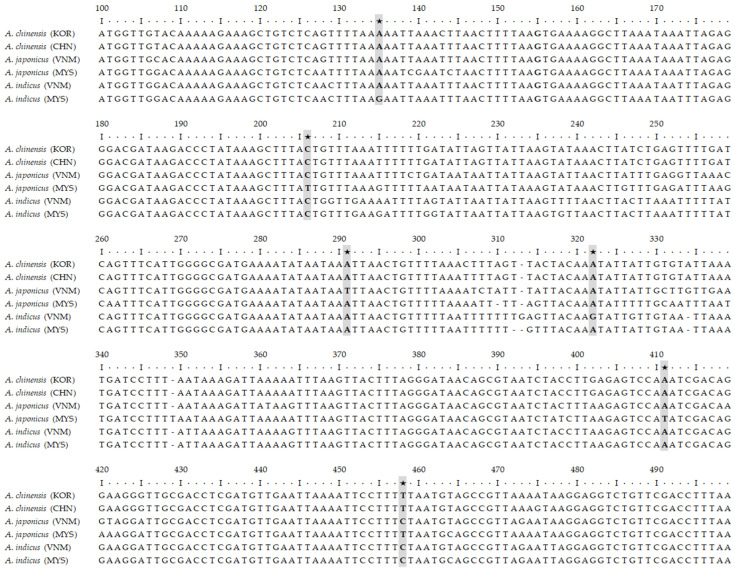
Candidate SNP marker sites selected from mitochondrial 16S rRNA region. The asterisks in the figure indicate the polymorphic sites for origin determination.

The haplotype diversity within groups of salted small-shrimp products classified by species and origin was confirmed by analyzing two mitochondrial gene regions. Subsequently, SNPs that can distinguish between the groups were identified. In the COI region, two candidate SNPs (sites 171 and 195) were identified (Figure 2), and in the 16S rRNA region, six candidate SNPs (sites 135, 206, 291, 322, 411, and 458) were identified (Figure 3). Using these candidate SNPs, the accuracy of distinguishing between groups was assessed. Within the *A. chinensis* group, only one SNP (COI-171) could distinguish between those of Korean and Chinese origin, whereas two or more SNPs were suitable for this purpose in all other groups (Table 2).

### 3.3. Designing SNP Genotyping Array

To facilitate genotyping using microarrays, marker development was performed for the selected SNP candidates using the Fluidigm D3 Assay Design Tool. Marker development was successfully completed for six candidates, excluding the 291 and 322 SNPs in the 16S rRNA region (Table 3). The two SNPs for which markers were not developed were found to be specific to *A. japonicus* and *A. indicus* from Vietnam, making it impossible to distinguish between them. Additionally, with the exclusion of the two markers, only one SNP (16S-458) was involved in discriminating between *A. chinensis* from China and *A. indicus* from Vietnam.

### 3.4. Analyzing Genotyping Data

Genotype information for six SNPs obtained from a total of 636 small-shrimp samples was used as training data for the ML analysis. The training data were composed of groups consisting of *A. chinensis* from Korea, *A. chinensis* from China, *A. japonicus* and *A. indicus* from Vietnam, and *A. japonicus* and *A. indicus* from Malaysia. The constructed training data were validated using the Random Forest algorithm with 80% as training data and 20% as test data, and achieved 90.65% accuracy. For Vietnamese *A. japonicas* and *A. indicus,* it was impossible to differentiate between the species due to them sharing the same SNP markers. However, the identification accuracy for other species and their origins was confirmed to be 100%.

Subsequently, salted small-shrimp samples were randomly collected for analysis, including 16 samples each from Korea, China, and Vietnam, and 32 samples from Malaysia. These samples were subjected to Fluidigm chip analysis. The results confirmed a 100% call rate for all samples, and the obtained genotype information was used as test data to perform species and origin identification through ML analysis. Korean salted small-shrimp samples were identified as 100% (16/16) Korean *A. chinensis*, and Chinese samples were also identified as 100% (16/16) Chinese *A. chinensis*. The Vietnamese samples identified as *A. japonicus* and *A.* indicus matched 100% (32/32) with the same origin. The Malaysian products matched 21.9% (7/32) with *A. japonicus* and 78.1% (25/32) with *A. indicus* (Table 4).

## 4. Discussion

The integrity of the seafood industry is critically dependent on accurate origin determination, a concern that has become increasingly significant amid rising consumer awareness about food safety and quality [29]. This study was performed to develop a reliable method to discriminate the origin of small shrimp, specifically targeting the species *A. chinensis*, *A. japonicus*, and *A. indicus*, which are commonly used in Korean cuisine. ML-based SNP analysis was performed to address issues related to mislabeling and ensure that consumers receive accurate information.

The results of the mtDNA analysis of the COI region and 16S rRNA region provided genetic distinctions among small-shrimp species and their geographical origins. Notably, *A. chinensis* from Korea and China exhibited significant genetic differences, as did *A. japonicus* and *A. indicus* from Vietnam and Malaysia. These genetic markers are critical for accurately identifying the provenance of small-shrimp products. Through SNP discovery, crucial genetic variations capable of differentiating between species and origins were identified. Specifically, eight candidate SNPs were identified in the COI and 16S rRNA regions. Six of these were successfully developed into markers using the Fluidigm D3 Assay Design Tool. These markers were instrumental in the genotyping process, which involved the analysis of a large number of shrimp samples to establish a robust database for ML.

The application of ML, in particular, supervised learning, showed high accuracy in classifying the origin of shrimp samples. The training data, derived from 636 small-shrimp samples, showed 100% accuracy in identifying the origin of test samples. This high level of precision underscored the potential of combining SNP analysis with ML for effective seafood origin determination. However, there are still challenges in identifying interspecific differences in Vietnamese samples. This remains a task to be addressed in future research.

The findings of this study have important implications for the seafood industry. Accurate origin determination is essential for maintaining consumer trust. By ensuring that products are correctly labeled, food fraud can be prevented and consumers can be protected from potential health risks associated with mislabeled seafood [30]. The method developed here provides a robust means for regulatory bodies to enforce labeling laws and standards. This can help to establish order in the distribution of seafood products and deter unscrupulous practices in the industry. For producers, especially those in regions with a reputation for high-quality seafood, accurate labeling can justify premium pricing. Highlighting the origin of products, such as Korean *A. chinensis* used in kimchi, can enhance market competitiveness and consumer preference.

However, as the countries of origin for imports become more diverse, the range of target species requiring additional analysis may increase. To address this issue, it will be necessary to expand the data set by including additional samples to improve the accuracy and performance of the analysis through continuous monitoring.

Overall, this study underscores the importance of accurate seafood origin determination for ensuring food safety, maintaining consumer trust, and supporting regulatory compliance. The successful integration of SNP analysis with ML provides a powerful means of determining the origins of small shrimp, addressing the challenges posed by mislabeling. As the seafood industry continues to evolve, such innovative approaches will be crucial for safeguarding the integrity of food products and promoting sustainable practices. Continued research and technological advances will be required to expand these methods to a broader range of species and regions, ultimately benefiting consumers, producers, and regulators.

## 5. Conclusions

This study provides insights into the species and origins of salted small-shrimp products from Korea, China, Vietnam, and Malaysia through genetic analysis. The results confirmed that Korean and Chinese small shrimp belonged to the same species (*A. chinensis*), but showed differences in some nucleotide variations. Similarly, Vietnamese and Malaysian small shrimp were identified as the same species (*A. japonicus* and *A. indicus*), with genetic differences based on their origins. These genetic variations are considered to result from genetic fixation due to geographic isolation. Based on these genetic differences, this study developed genetic markers and used ML analysis to establish a method for distinguishing the origins of small shrimp. The findings underscore the value of genetic analysis for accurately identifying small-shrimp species in commercial products and revealing potential variations based on origin. In addition, this method can be applied across various fields and will contribute to food distribution safety.

## Figures and Tables

**Table 1:** The information of salted small-shrimp products used in this study.

Sample No.	Indicated Origin		Collection Date	Population Size
1	Korea	Sinan-gun	7 March 2023	48
2	Korea	-	7 March 2023	48
3	Korea	Ganghwa-gun	7 March 2023	47
4	Korea	-	8 March 2023	48
5	Korea	Incheon-si	24 March 2023	48
6	Korea	Yeonpyeong Island	24 March 2023	43
7	Korea	Boryeong-si	24 March 2023	48
8	Korea	Taean-gun	24 March 2023	47
9	Korea	-	24 March 2023	48
10	Korea	Mokpo-si	24 March 2023	48
11	China	-	24 March 2023	46
12	Vietnam	-	7 March 2023	46
13	Vietnam	-	7 March 2023	48
14	Malysia	-	26 June 2023	23

**Table 2:** Candidate SNP marker information for identifying origin of small shrimp.

Species (Origin)	COI Region		16S rRNA Region					
	171	195	135	206	291	322	411	458
*A. chinensis* (KOR)	C	T	A	C	A	A	A	T
*A. chinensis* (CHN)	T	T	A	C	A	A	A	T
*A. japonicus* (VNM)	T	T	A	C	T	A	A	C
*A. japonicus* (MYS)	-	-	A	T	A	A	T	T
*A. indicus* (VNM)	T	T	A	C	A	G	A	C
*A. indicus* (MYS)	T	C	G	C	A	A	A	C

**Table 3:** A list of Fluidigm SNP-type assays developed in this study.

SNP ID	Target Gene	Position	SNP	Fluidigm Assay ID
COI-171	COI	171	..CCAGA[C/T]ATAGC..	GTA0343170
COI-195	COI	195	..AATAA[C/T]ATAAG..	GTA0343171
16S-135	16S rRNA	135	..TTTAA[G/A]AATTA..	GTA0343174
16S-206	16S rRNA	206	..CTTTA[T/C]TGTTT..	GTA0343420
16S-417	16S rRNA	411	..GTCCA[T/A]ATCGA..	GTA0343172
16S-464	16S rRNA	458	…CCTTT[T/C]TAATG..	GTA0343173

**Table 4:** The accuracy of small-shrimp origin identification using machine learning analysis with 6 SNP markers.

Species (Origin)	Sample 1 (Korea)	Sample 2 (China)	Sample 3 (Vietnam 1)	Sample 4 (Vietnam 2)	Sample 5 (Malaysia)
*A. chinensis* (KOR)	16	-	-	-	-
*A. chinensis* (CHN)	-	16	-	-	-
*A. japonicus* (VNM)	-	-	16	16	-
*A. japonicus* (MYS)	-	-	-	-	7
*A. indicus* (VNM)	-	-	-	-	-
*A. indicus* (MYS)	-	-	-	-	25
Species accuracy	100%	100%	100%	0%	100%
Origin accuracy	100%	100%	100%	100%	100%

## Data Availability

The data sets generated and/or analyzed in the present study are available from the corresponding author upon reasonable request.
